# Can the perceived risk of particulate matter change people's desires and behavior intentions?

**DOI:** 10.3389/fpubh.2022.1035174

**Published:** 2022-11-16

**Authors:** Junghyun Park, Yunmi Park, Jae Leame Yoo, Gong Yue, Jongsik Yu

**Affiliations:** ^1^College of Hospitality and Tourism Management, Sejong University, Seoul, South Korea; ^2^Department of Aviation Service, Cheongju University, Cheongju-si, South Korea; ^3^Department of Aeronautical Science and Flight Operation, Cheongju University, Cheongju-si, South Korea; ^4^Business School Tourism and Hospitality Management, Xuzhou University of Technology, Xuzhou, China; ^5^Department of Hotel and Foodservice Management, Cheongju University, Cheongju-si, South Korea

**Keywords:** particulate matter, perceived risks, TPB, desire, behavior intention

## Abstract

Particulate matter (PM) is a hazardous airborne pollutant that encompasses all airborne particles with diameters ranging from 0.001 to 100 μm. It is composed of total suspended particles (TSPs), consisting of two main particle sizes: PM10 and PM2.5. PM poses various threats to human health because of its rapid mobility and its ability to spread over a wide area. In particular, it has long-term negative effects on such organs as the lungs and heart. China and South Korea, located in Northeast Asia, are representative of the countries at risk of PM, and their populations live with an awareness that the harms of PM go beyond physical risks. Therefore, based on previous studies, this study classifies the perceived PM risks into physical, psychological, financial, functional, and time risks. It has tried to verify the effect of this risk perception on the behavior intention of Chinese and Koreans and examine the moderating effect according to the difference in nationality. The study's conceptual model was constructed by applying Ajzen's proven theory of planned action. Utilizing AMOS 22.0 and SPSS 22.0, an analysis was performed. Following this analysis, it was determined that there was a significant causal relationship between perceived PM risk and behavioral attitudes, subjective norms, and perceived behavioral control. Additionally, it was discovered that perceived PM risk significantly impacted desire and behavioral intention. These findings demonstrate that when persons are exposed to high concentrations of PM, they perceive a variety of risks that go beyond the merely physical, and they can form different attitudes depending on their nationality. This study greatly contributes to the theoretical and practical implications by presenting more diverse perspectives on PM risk.

## Introduction

Rapid economic growth and industrialization have created a new social problem and threat to human health in the form of particulate matter (PM). PM is a dangerous airborne pollutant that encompasses all airborne particles whose diameters range from 0.001 to 100 μm (1). PM is composed of total suspended particles (TSPs), consisting of two main particle sizes: PM10 and PM2.5. PM10, and PM2.5 are very different from the traditional dust that can be generally observed around us, and they can be categorized into fine dust and ultra-fine dust according to their diameters. PM10, which we call “fine dust,” is dust smaller than 10/1,000 mm, and PM2.5, which we call “ultra-fine dust,” is dust smaller than 2.5/1,000 mm, less than 1/20th to 1/30th the diameter of a human hair (about 60 μm). Only an electron microscope can detect it (1, 2). This fine PM may be much more common than we think. Its primary sources are vehicles, power plants, and such activities as burning coal and wood for fuel. Natural disasters, such as natural or man-made wildfires, can also release PM into the atmosphere (3). According to one misconception, fine PM only affects the quality of outdoor air, but it is also prevalent in homes. PM can be added to our home through routine activities like cooking (particularly frying, stir-frying, broiling fish, etc.), smoking, or using a wood stove or other household fuel (3). These fine PM particles can also easily travel long distances, which can negatively affect the surrounding area for miles. In particular, they adversely affect human activity and the body (4, 5).

Fine PM is only one-fifth the size of a human hair. Therefore, it is highly likely to seep into the body without being filtered out of the nose or bronchi. The fine PM that enters the body and penetrates the lungs causes asthma and lung diseases, as well as inflammation due to the action of immune cells to remove it (2, 4, 5). Ultra-fine PM has a larger surface area than fine PM; more harmful substances can be adsorbed and are more likely to penetrate blood vessels and move to other human organs (3). The WHO estimates that in 2016, ~58% of premature deaths from outdoor air pollution were related to stroke or ischemic heart disease, while 6% of deaths were attributable to lung cancer, and 18% were brought on by chronic obstructive pulmonary disease brought on by acute lower respiratory infections (4). Public awareness of the risk of respiratory and lung diseases caused by fine PM has also limited human activities (6–8). Warning messages from the government and media, such as about refraining from outdoor activities and recommending masks due to high fine PM concentrations, have raised awareness of negative risks and anxiety about outdoor activities and become an important negative catalyst for reducing outdoor activities and walking time (7–9).

Particulate matter is a global problem, affecting various regions in various ways. For decades, Northeast Asian countries have suffered from severe PM concentrations (7, 9–11). In particular, high PM concentrations in China and Korea are causing great concern, and public interest in their health problems and poor quality of life and the related risks is gradually increasing (8, 10, 11). China has experienced rapid dynamics and demographic changes because of the speed of its economic development over the past few decades. This has coincided with an increase in PM levels due to increased energy emissions and industrial waste. In particular, PM 2.5, a major health burden, plays an increasingly negative role in China's social and economic development (12). Fine PM concentrations in China have exceeded WHO air quality standards and epidemiological data demonstrate a persistent link between rising respiratory and cardiovascular disease rates and PM concentrations (8, 13). Korea is not much different from China. In instance, Korea placed first among OECD nations for PM2.5 concentration (24.8 g/m^3^/year) in 2019, and 61 Korean cities were listed among the top 100 cities with high levels of urban pollution, making up the largest share among OECD members (14). The Korean government is responding to the PM problem by strengthening environmental standards and enacting the “Special Act on the Reduction and Management of Fine Dust” law in 2016 (7, 15). However, the perceived risk (i.e., physical, psychological, financial, functional, and time) of PM that has accumulated in a relatively short period is affecting people's lives in various ways (6).

In addition to the negative effects on health, high PM concentrations reduce urban residents' outings and interactions, decreasing consumption and reducing overall vitality (16, 17). Specifically, when PM accumulates, people reduce such outdoor leisure activities as jogging, walking, cycling, and hiking, and workers' productivity declines, which has a negative economic impact (16, 18, 19). Health costs rise due to various diseases associated with PM (16). Braithwaite et al. (20) and Kim et al. (21) found that high fine dust levels negatively affect psychological and mental health, increasing suicide rates and lowering the overall quality of life. Therefore, PM poses various threats to human life. Nevertheless, studies have only focused on the health effects of PM, so a broader perspective is necessary. Insight is also needed into how awareness of this risk of PM can change people's cognitive behavior. Therefore, this study investigates how this awareness can change human desires and behavioral intentions based on Ajzen's (22) theory of planned behavior (TPB), which has been proven on reasoned human actions.

The following queries are specifically addressed in this study: (1) among the perceived PM risks presented in this study, which risk is the most widely recognized; (2) does PM risk perception affect sub-factors of TPB and behavior intention through Ajzen's verified theory of TPB; (3) does the perception and behavior of PM risk differ according to nationality (China and Korea)?

## Literature review

### Characteristics and perceived risks of PM

One of the major global environmental issues is air pollution. Worldwide, an average of over 7 million people die prematurely each year from breathing air containing a high concentration of pollutants (23). The main substances polluting the air are persistent organic pollutants, fine dust, heavy metals, nitrogen oxides, and carbon monoxide (24). Especially harmful to humans is PM, which can have serious effects (4, 5, 25). PM can be classified according to particle size and is generally categorized into PM10 and PM2.5. The potential for health issues to be caused by the particles is directly correlated with their size. Exposure to these particles can affect both the lungs and the heart. According to WHO surveys, an increase of 10 μg/m^3^ in PM10 levels is generally correlated with a 0.2–0.6% increase in daily mortality, and long-term exposure to PM2.5 increases the risk of cardiopulmonary death per 10 μg/m^3^ of PM by 6–13% (23). Consequently, people are concerned about the health damage caused by PM, so they change their activity patterns, such as by refraining from outdoor activities (16–18). PM also leads to psychological fear and physical risk for workers (16, 19), which lowers their productivity and entails financial risk, psychological risk, and negative consequences such as drugs or suicide (21, 26). Therefore, people can experience various risks through PM, and the anxiety consumers feel about the uncertain results that may arise can be defined as perceived risk (27, 28).

Perceived risk refers to the psychology behind people's behavior as it reflects uncertainty toward the future. These uncertainties directly affect people's behavioral responses (29–31). The term “perceived risk” also refers to how someone feels and comprehends the many externally present threats, emphasizing the impact of personal experiences on hunches and subjective impressions (31). This risk awareness is fundamental in psychological and physical risk environments, as it determines which risks people are concerned with and how they respond to them (31, 32). Perceived risk can be categorized according to several dimensions, measured through various indicators (28). Stone and Grønhaug (33) found that perceived risk includes physical, psychological, financial, social, functional, and time risks. In examining green consumer electronic products, Pathak and Pathak (34) classified the perceived risk into five sub-dimensions, physical, psychological, financial, functional, and time, and verified the significance of each. In examining the perceived risk and psychological distance of air pollution, Liu et al. (30) showed that people could perceive physical, psychological, economic, and functional risks according to air pollution. Therefore, this study presents physical, psychological, financial, functional, and time risks as sub-factors of the perceived risk of PM and attempts to verify the effect.

#### Physical risk

In the environmental sector, physical risks are often characterized as dangers brought on by the physical consequences of environmental deterioration and climate change (35). Environmental destruction poses various threats, but the most critical issue these days is the risk caused by PM. PM shortens the lifespan of the entire body, beginning with the fetus and continuing until death, which causes premature mortality, primarily due to respiratory and cardiovascular problems (2, 4, 12). The everyday consequences of PM are becoming increasingly serious and severely threatening our health (2–4). Exposure to PM can lead to premature death from cardiovascular disease (arrhythmia, heart attack) and respiratory diseases (asthma attack, bronchitis, pneumonia) (2, 4). In addition to the negative effects on health, PM accumulates, increases such as hospital visits, hospitalizations, absences from school and work, and limited outdoor activities. Yan et al. (16) and Sass et al. (26) found that in areas with high levels of PM, the perceived risk to the body tends to lead people to reduce the amount of time they spend outdoors. Their research has also shown that high levels of PM harm the pathogenesis of the brain and psychiatric disorders (3, 26) and entail an exceptionally high risk for the elderly and children. However, PM has also been proven to affect healthy people (3).

#### Psychological risk

Psychological risk is the possibility of a psychological injury occurring when a person is exposed to a hazard. From a psychological standpoint, hazards are instances or elements that might raise the possibility of a stress response, essentially a bodily, psychological, or emotional reaction (36). A higher risk of chronic disease and mortality is linked to psychological discomfort, which can also affect everyday living activities and social interaction. People tend to spend less time outdoors in areas with high PM levels. This avoidance behavior reduces the number of times people are exposed to sunlight, which increases the likelihood that vitamin D deficiency will cause or exacerbate psychological risk (26, 37). In addition, high PM concentration levels negatively affect psychologically such as increased anxiety, psychosis, perceived stress, depression, and suicide rates (20, 21). In this context, Sass et al. (26) found that increased psychological anguish is significantly linked to PM2.5. Additionally, Clifford et al. (38) and Luo et al. (39) also found a significant relationship between PM and anxiety, suicide, and depression.

#### Financial risk

Financial risk is the potential for monetary loss on an investment or business venture (40, 41). Climate change poses major risks that are already affecting the lives and finances of many people. PM has a complex relationship with climate change. PM can have either warming or cooling effects on the climate (42). Global insurance firm Marsh McLennan estimates that climate change will put about 2% of global financial assets at risk by 2100 (43). Therefore, PM, which plays an essential role in climate change, may entail various financial risks. A high level of PM reduces people's consumption activities. It affects sales of nearby stores, which in turn has a dangerous effect on the finances of the companies that produce the products sold. Additionally, it causes a rise in prices due to decreased production of agricultural and fishery products and generates additional expenditures due to the installation of air purifiers at home, further purchases of masks, and increased use of delivery food. Guo et al. (44) found a positive correlation between the air quality index (AQI) of some Chinese cities and the stock price return of local companies.

#### Functional risk

Perceived risks may include functional risks, which may raise fears and suspicions that external hazards will prevent people from conducting a plan or activity. People are restricted from outdoor activities due to particulate matter, and implementing policies such as government measures to reduce PM also limits the use of vehicles. Regarding physical functional risk, long-term exposure to airborne particulates leads to cognitive decline. A Chinese study found that exposure to PM reduced cognitive abilities in speech and math tests (45). Additionally, high levels of PM impair cognitive function in decision-making and reduce productivity and worker performance (39). Kahn and Li (46) also found that prolonged exposure to PM increased the risk of death and lowered the emotional wellbeing and productivity of outdoor workers.

#### Time risk

Perceived risks from PM may also include time risks (6, 30, 33). Time risk refers to wasted or lost time as a result of bad decisions or unexpected circumstances. Purchasing an item is a representative example of time risk. Time risk involved includes navigation and/or submitting orders to buy, waiting for delivery of the item to home, and returning it for a replacement because the product does not meet consumer expectations (47–49). If we apply this to the PM risk, the time required to purchase and install an air purifier due to deteriorating air quality and the time it takes for regular maintenance can be perceived as time risks. Other examples of time risks are reducing leisure and economic activity (work) hours, changing business schedules due to high PM concentration, using public transportation to lower pollution levels, and purchasing masks to prevent health problems (49, 50). Therefore, this perception of time risk has been found to have a negative effect on people's behavior and intentions (48).

### Theory of planned behavior

The TPB is a psychological theory that links beliefs to behavior. Ajzen (22) developed TPB to increase the predictive capacity of the theory of reasoned action (TRA). TPB was intended to incorporate perceived behavioral control. TPB views individuals as rational decision-makers who weigh all possible outcomes before making a choice (22). The theory contends that three fundamental variables—attitude, subjective norms, and perceived behavioral control—significantly impact a person's behavioral intentions (22). Attitude, one of the constituent factors of TPB, indicates how an individual evaluates a given behavior, whether positively or negatively, and predicts the intention to perform a protective behavior (22, 51). Subjective norms manifest social influence and are defined as the extent to which people feel pressured or not to perform (or not) an action by significant others (51). Perceived behavioral control can be described as “an individual's beliefs about how easy or difficult the performance of a behavior is.” (22). The most proximal predictor of human social behavior, in turn, is the behavioral intention, which is a core principle of TPB (22). Behavioral intentions represent upcoming behaviors people expect and prepare for (52). They show expectations associated with particular behavior in a specific mechanism and can be considered to predict the likelihood that the behavior will be performed (22, 51, 52). Therefore, the TPB has been used in several academic disciplines to understand better the processes by which conscious decisions are made, particularly concerning environmental and health risks (53).

Perceived risks are fundamental to environmental and health risk communication, as they determine which risks people are concerned with and how they respond (27). An individual's social, cultural, and situational factors influence that individual's perceptions and are significantly causally related to attitudes, behaviors, and perceived behavior control (27, 54). Fishbein and Yzer (55) suggested that risk perception can be conceptualized as a terminal predictor of behavioral intention. Risk perception can be regarded as an essential determinant of behavioral intention through subjective norms (56). When individuals engage in a certain behavior, risk perception interacts with perceived behavioral control, and an individual's risk perception increases their desire to act according to their sense of self-efficacy to engage in preventive behavior (57). People with higher risk perception are more responsive to behavioral consequences (attitudes) to form positive intentions. According to an air pollution study, persons who experience adverse health effects from PM evaluate the risk as more severe, have higher environmental standards and are more inclined to act to lessen the risk (57, 58). In particular, direct experience with risk minimizes an individual's psychological distance from risk and encourages risk mitigation measures (59). Psychological distance is an individual's perception that affects behavior, and the farther the psychological distance to the environment, the less eco-friendly intention, and the closer the psychological distance to the environment, the more positive to support and participation in pro-environmental behavior (30). Thus, when people perceive the various dangers of PM, they can increase their desire and intention to perform specific behaviors while reducing their psychological distance. In particular, an individual's normative behavior toward the perceived risk of PM significantly affects the intention to take preventive actions under the influence of surrounding groups and social factors (60–62). In light of previous research, the following hypotheses are put forth:

Hypothesis 1: The perceived risk of PM negatively affects attitude toward the behavior.

Hypothesis 2: The perceived risk of PM negatively affects the subjective norm.

Hypothesis 3: The perceived risk of PM negatively affects perceived behavioral control.

Hypothesis 4: The attitude toward the behavior positively affects desire.

Hypothesis 5: The subjective norm positively affects desire.

Hypothesis 6: The perceived behavioral control positively affects desire.

Hypothesis 7: The desire positively affects behavior intention.

### PM perception in China and Korea

Like many other nations that experienced rapid industrialization, China and Korea experienced strong economic growth and severe pollution before concerted attempts were made to improve air quality (9–11). China, in particular, has seen significant increases in energy consumption and air pollution emissions due to rapid economic growth. As a result, fine PM pollution is one of the most critical environmental problems in modern-day China, especially in highly industrialized urban areas (10, 11, 59). Given the many threats that PM demonstrably poses to human health, the Chinese public has perceived the risks posed by air pollution as being more severe (11, 63, 64). This is because regular haze pollution has increased public anxiety in China and raised the possibility of civil instability (64). Lee (65) established that Chinese adolescents' risk perception toward environmental messages (e.g., PM severity) positively affected environmental attitudes and shaped their environmental desire and behavior intentions. Yan and Wu (53) also found that public perception of PM risks significantly affected their attitudes, norms, and behavioral intentions.

According to the Korean National Institute of Environmental Research, Seoul, Korea, is one of the world's most polluted cities. Seoul's mean PM10 levels were higher between 2009 and 2013 than in several of the world's biggest cities, including Los Angeles, Tokyo, Paris, and London (66). In addition, in 2019, its PM2.5 concentration (24.8 μg/m^3^/year) was the highest among OECD nations (7). Korean interest in PM increased after the 1988 Seoul Olympics, as the government concentrated on the air pollution condition, remedial actions, and anticipated risk levels based on the season (7, 67). In particular, people had a greater perception of PM risks with increased media attention and warnings about outdoor activities and masks (67). In addition, the perceived risk of PM differed depending on the region. Kim et al. (7) and Whitmarsh (58) found a correlation between residency in areas with severe PM and negative attitudes and behavioral intentions. Furthermore, the risk perception of air pollution significantly affects self-protection through attitudes, perceived norms, and self-efficacy (68).

Public perception of PM influences its emotional and behavioral responses (69). Research has found that if the AQI rises by 100 points on a given day, “emigration” searches will increase by roughly 2.3–4.8% the next day. In addition, such an effect is more pronounced when the AQI level is above 200; the effect differs according to the destination country and metropolitan area (69). In other words, numerous variables, such as gender, family income, region, age, education level, personal experiences, and health symptoms, may impact how each individual perceives air pollution (63). In addition, the public perception of PM is determined by social networks, media, and other sociocultural factors, which are highly localized (70). Considering that individuals may have different perceptions of risk depending on the degree and level of PM, their perceptions and behavior intentions may differ depending on whether they live in China or Korea. In light of previous research, the following hypotheses are put forth:

Hypothesis 8a: Nationality moderates the relationship between the perceived risk of PM and attitude toward behavior.

Hypothesis 8b: Nationality moderates the relationship between the perceived risk of PM and subjective norm.

Hypothesis 8c: Nationality moderates the relationship between the perceived risk of PM and perceived behavioral control.

Hypothesis 8d: Nationality moderates the relationship between attitude toward behavior and desire.

Hypothesis 8e: Nationality moderates the relationship between subjective norm and desire.

Hypothesis 8f: Nationality moderates the relationship between perceived behavioral control and desire.

Hypothesis 8g: Nationality moderates the relationship between desire and behavior intention.

### Research model

**Figure 1:**
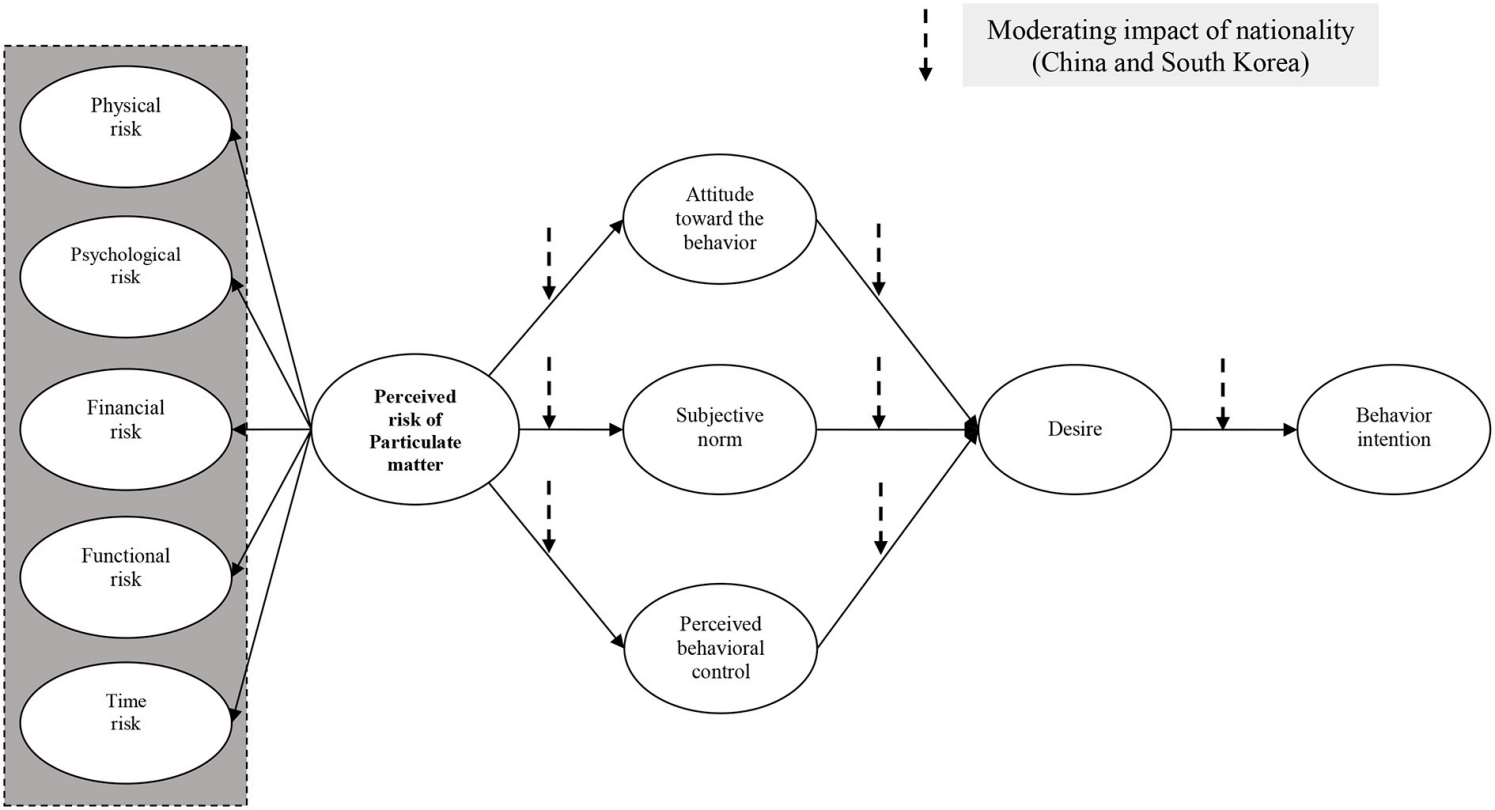
Proposed conceptual framework.

The 12 theoretical components that make up this study's conceptual framework explain how the perceived risk of PM affects people's behavioral intentions. In the conceptual framework of the presented study, the moderating effect of nationality (China and Korea) is added to examine the difference. In this study, the perceived risks of PM are classified into physical, psychological, financial, functional, and time risks. The suggested theoretical framework has a total of eight hypotheses. Figure 1 displays the research model that this study presents.

## Methodology

### Measurement tools

In this study, the perceived PM risk (e.g., physical, psychological, financial, functional, time), Attitude through Ajzen (22)'s Theory of Planned Behavior (TPB), using measurement items whose reliability and validity have been verified in prior research. The attitude toward the behavior, subjective norm, perceived behavioral control, desire, and behavioral intention were measured. Specifically, three questions were used for each of the five risks presented to measure the perceived risk of PM based on Dayour et al. (71); Quintal et al. (72). Also, according to Han (73), Perugini and Bagozzi (74), Ajzen (22), and Han et al. (75), each of the three questions measured attitude toward the behavior, subjective norm, perceived behavioral control, and desire. Finally, according to Han and Yoon's (76) research, three questions were used to measure behavioral intention. All of the questionnaires used in this study were multi-item, and the respondents were people living in areas with high PM concentrations in China and Korea. Responses were rated on a 7-point Likert scale ranging from 1 to 7 points (strongly disagree to strongly agree, respectively). In addition, a preliminary test was conducted so that survey respondents could understand the survey content more clearly and revise and supplement the survey content. The preliminary test was conducted for university professors majoring in environmental engineering and graduate students majoring in atmospheric environmental engineering.

### Data collection and sample characteristics

In this study, a sample was selected through the web-based system of a data collection institution, and the collected questionnaire was used for analysis. The respondents were randomly chosen via email from those who experienced PM in China and Korea. A total of 322 persons were recruited through this method, and 318 persons were included in the empirical analysis; four individuals were excluded for insincere answers. Using SPSS 22.0, frequency analysis was performed to determine the demographic details of the sample. Specifically, the following are the demographic details of the survey respondents: By nationality, 42.1% were Chinese, and 57.9% were Korean. By gender, 48.1% were male, and 51.9% were female; for age, 33% were under the age of 20, 46.5% were in their 30s, 17% were in their 40s, and 3.5% were over the age of 50; in terms of annual income, 13.5% had less than US$30,000, 81.8% had between US$30,000 and US$50,000, 4.1% had between US$50,000 and US$70,000, and 0.6% earned more than US$70,000; regarding academic background, 7.2% of respondents had a college degree, 82.4% had a bachelor's, and 10.4% had a master's or higher; regarding marriage, 63.8% were single, and 36.2% were married.

## Results

### Presented measurement model results

Confirmatory factor analysis (CFA) was performed in this study to assess the validity and reliability of the proposed research model. CFA is the most practical analysis technique for confirming the validity and one-dimensionality of the scale, as well as the reliability of the measurement model (77). The analysis results through CFA are as follows. The statistical validity of the measurement model used in this investigation was satisfactory with χ^2^ = 944.111, df = 360, *p* < 0.01, χ^2^/df = 2.623, RMSEA = 0.072, CFI = 0.935, TLI = 0.921. Standardized regression weights were then measured to confirm the reliability of the measured properties. Results ranged from 0.648 to 0.925. Therefore, reliability was confirmed, exceeding the standardized regression weight of 0.5 for all assessed attributes. The average variance extracted (AVE) and composite reliability (CR) values were analyzed to verify the proposed measurement variables' internal consistency and central validity. As a result, AVE values arranged from 0.652 to 0.723, and CR values ranged from 0.849 to 0.887. As a result, it can be concluded that the measured attributes have acceptable internal consistency and central validity. Finally, discriminant validity was examined to confirm that the proposed notions could be distinguished. When the AVE value is greater than the square of the correlation coefficient, the discriminant validity can be irreproachable (78). Because the AVE value was greater than the correlation coefficient's square value, the analysis's findings supported discriminant validity. The results of CFA are presented in Table 1.

**Table 1:** Presented measurement model results.

	**(1)**	**(2)**	**(3)**	**(4)**	**(5)**	**(6)**	**(7)**	**(8)**	**(9)**	**(10)**
Physical risk (1)	1.000									
Psychological risk (2)	0.651^a^ (0.423)^b^	1.000								
Financial risk (3)	0.621 (0.385)	0.522 (0.272)	1.000							
Functional risk (4)	0.592 (0.350)	0.532 (0.283)	0.671 (0.450)	1.000						
Time risk (5)	0.620 (0.384)	0.539 (0.290)	0.427 (0.393)	0.619 (0.383)	1.000					
Attitude toward the behavior (6)	−0.407 (0.165)	−0.452 (0.204)	−0.522 (0.272)	−0.534 (0.285)	−0.638 (0.407)	1.000				
Subjective norm (7)	−0.422 (0.178)	−0.472 (0.222)	−0.588 (0.345)	−0.625 (0.390)	−0.477 (0.227)	0.550 (0.302)	1.000			
Perceived behavior control (8)	−0.484 (0.234)	−0.487 (0.237)	−0.461 (0.212)	−0.595 (0.354)	−0.598 (0.357)	0.618 (0.381)	0.590 (0.348)	1.000		
Desire (9)	−0.335 (0.112)	−0.374 (0.139)	−0.608 (0.369)	−0.469 (0.219)	−0.616 (0.379)	0.585 (0.342)	0.591 (0.349)	0.589 (0.346)	1.000	
Behavior intention (10)	−0.366 (0.133)	−0.464 (0.218)	−0.492 (0.242)	−0.466 (0.217)	−0.536 (0.287)	0.505 (0.255)	0.484 (0.234)	0.580 (0.336)	0.620 (0.384)	1.000
Mean	5.024	4.693	5.090	5.169	4.860	2.711	2.967	2.656	2.812	4.715
SD	1.249	1.220	1.367	1.205	1.299	1.090	1.252	1.303	1.325	1.300
CR	0.855	0.880	0.853	0.857	0.849	0.887	0.856	0.880	0.860	0.886
AVE	0.662	0.710	0.660	0.667	0.652	0.723	0.664	0.710	0.672	0.721

Goodness-of-fit statistics for the measurement model: χ^2^ = 944.111, df = 360, p < 0.001, χ^2^/df = 2.623, RMSEA = 0.072, CFI = 0.935, NFI = 0.900, TLI = 0.921.

a Correlations between the variables are below the diagonal.

b The squared correlations between the variables are within the parentheses.

### Structural equation modeling

This study verified conceptual characteristics and proposed hypotheses using the maximum likelihood method through structural equations. The model fit for this study was found to be adequate with χ^2^ = 1141.166, *df* = 391, *p* < 0.01, χ^2^/*df* = 2.919, RMSEA = 0.078, CFI = 0.916, TLI = 0.907. Therefore, as a result of testing the lower five risk factors of perceived risk of PM presented in this study, it was found that people perceive all five risks as significantly high. The results of the initial seven hypotheses presented are as follows. As a result of testing hypotheses 1, 2, and 3, perceived risk of PM has a negative affect on attitude toward the behavior (β = 0.785, *p* < 0.01), subjective norm (β = 0.831, *p* < 0.01) and perceived behavioral control (β = 0.862, *p* < 0.01) was also found to have a negative effect. Therefore, hypotheses 1, 2, and 3 were supported. Next, the effect of attitude toward the behavior, subjective norm, and perceived behavioral control on desire was verified. As a result, attitude toward the behavior and desire (β = 0.292, *p* < 0.01), subjective norm and desire (β = 0.194, *p* < 0.05), perceived behavioral control and desire (β = 0.256, *p* < 0.01) were all significant appeared to have an impact. Therefore, hypotheses 4, 5, and 6 were all supported. Finally, the effect of desire on behavioral intention was tested. Desire was found to have a significant effect on behavioral intention (β = 0.633, *p* < 0.01). Therefore, hypothesis 7 was supported.

The use of a mediating framework can greatly help understand the complex relationships between study components within a theoretical model (58). Therefore, indirect effects were validated using bootstrap to help understand the complex relationships in the model. Following the analysis, the perceived risk of PM was determined by desire (β_Perceived risk of particulate matter → attitude toward the behavior &_ _subjective norm & perceived behavior control → desire_ = −0.612), behavior intention (β_Perceived risk of particulate matter → attitude_ _toward the behavior & subjective norm & perceived behavior control &_ _desire → behavior intention_ = −0.388) had a significant indirect effect. The components of TPB are attitude toward the behavior and behavior intention (β_Attitude toward the behavior → desire → behavior intention_ = 0.185), subjective norm and behavioral intention (β_Subjective norm → desire → behavior intention_ = 0.123), perceived behavioral control and behavior intention (β_Perceived behavior control → desire → behavior intention_ = 0.162), significant indirect effects were verified only between subjective norm and behavioral intention. Therefore, the mediating role of attitude toward the behavior, subjective norm, perceived behavior control, and desire within the theoretical framework presented in this study was partially demonstrated. Table 2 shows the results of these analyzes.

**Table 2:** Structural equation modeling.

**Hypothesized paths**		**Coefficients**	***t*-values**
H1: Perceived risk of particulate matter	→ Attitude toward the behavior	−0.785	−12.540^**^
H2: Perceived risk of particulate matter	→ Subjective norm	−0.831	−13.724^**^
H3: Perceived risk of particulate matter	→ Perceived behavior control	−0.862	−14.765^**^
H4: Attitude toward the behavior	→ Desire	0.292	3.973^**^
H5: Subjective norm	→ Desire	0.194	2.489^*^
H6: Perceived behavior control	→ Desire	0.256	3.209^**^
H7: Desire	→ Behavior intention	0.633	11.186^**^
Indirect effect:		Explained variance:	
β_Perceived risk of particulate matter → attitude toward the behavior & subjective norm & perceived behavior control → desire_ = −0.612^**^ β_Perceived risk of particulate matter → attitude toward the behavior & subjective norm & perceived behavior control & desire → behavior intention_ = −0.388^**^ β_Attitude toward the behavior → desire → behavior intention_ = 0.185^**^ β_Subjective norm → desire → behavior intention_ = 0.123 β_Perceived behavior control → desire → behavior intention_ = 0.162		*R*^2^ (physical risk) = 0.723 *R*^2^ (psychological risk) = 0.730 *R*^2^ (financial risk) = 0.698 *R*^2^ (functional risk) = 0.911 *R*^2^ (time risk) = 0.741 *R*^2^ (attitude toward the behavior) = 0.617 *R*^2^ (subjective norm) = 0.691 *R*^2^ (perceived behavior control) = 0.743 *R*^2^ (desire) = 0.436 *R*^2^ (behavior intention) = 0.401	

* *p* < 0.05,

** *p* < 0.01.

Goodness-of-fit statistics for the measurement model: χ^2^ = 1141.166, df = 391, *p* < 0.001, χ^2^/df = 2.919, RMSEA = 0.078, CFI = 0.916, IFI = 0.917, TLI = 0.907.

### Structural invariance model assessment

**Figure 2:**
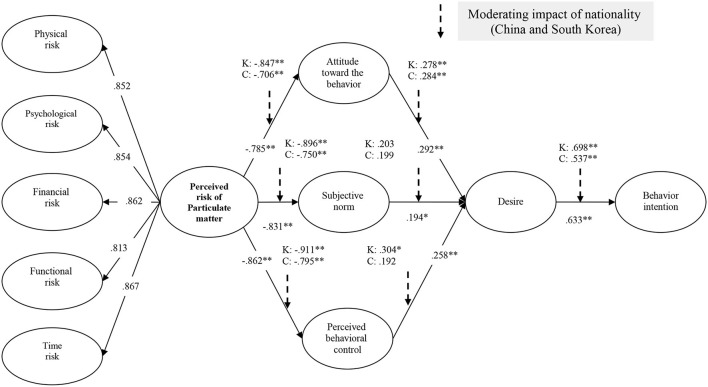
Results of structural model.

The proposed research model for the effect of perceived PM risk on behavioral intentions conducted an invariance test to identify the moderating roles according to nationality (China and Korea). Therefore, the proposed hypothesis H8a–g was tested by dividing it into a Chinese group (*n* = 134) and a Korean group (*n* = 184). Analysis revealed that nationality differences in the relationship between perceived risk of PM and attitude toward the behavior [Δχ2 (1) = 4.812, *p* < 0.05] play a significant moderating role. However, perceived risk of PM and subjective norm [Δχ2 (1) = 2.784, *p* > 0.05], perceived risk of PM and perceived behavior control [Δχ2 (1) = 2.729, *p* > 0.05], attitude toward the behavior and desire [Δχ2 (1) = 0.158, *p* > 0.05], subjective norm and desire [Δχ2 (1) = 0.009, *p* > 0.05], perceived behavior control and desire [Δχ2 (1) = 0.123, *p* > 0.05], nationality differences in the relationship between desire and behavior intention [Δχ2 (1) = 2.998, *p* > 0.05] does not play a significant moderating role. Therefore, only H8a is accepted, and all H8b–H8g are rejected. Detailed results are shown in Table 3 and Figure 2.

**Table 3:** Structural invariance model assessment.

**Paths**	**Korean (*n* = 184)**	**Chinese (*n* = 134)**	**Baseline model (freely estimated)**	**Nested model (constrained to be equal)**
	**β**	**β**		
H8a: Perceived risk of particulate matter → attitude toward the behavior	−0.847^**^	−0.706^**^	χ^2^ (806) = 1699.560	χ^2^ (807) = 1704.372^a^
H8b: Perceived risk of particulate matter → subjective norm	−0.896^**^	−0.750^**^	χ^2^ (806) = 1699.560	χ^2^ (807) = 1702.344^b^
H8c: Perceived risk of particulate matter → perceived behavior control	−0.911^**^	−0.795^**^	χ^2^ (806) = 1699.560	χ^2^ (807) = 1702.289^c^
H8d: Attitude toward the behavior → desire	0.278^**^	0.284^**^	χ^2^ (806) = 1699.560	χ^2^ (807) = 1699.718^d^
H8e: Subjective norm → desire	0.203	0.199	χ^2^ (806) = 1699.560	χ^2^ (807) = 1699.569^e^
H8f: Perceived behavior control → desire	0.304^*^	0.192	χ^2^ (806) = 1699.560	χ^2^ (807) = 1699.683^f^
H8g: Desire → behavior intention	0.698^**^	0.537^**^	χ^2^ (806) = 1699.560	χ^2^ (807) = 1702.558^g^
Chi-square test: ^a^ Δχ^2^ (1) = 4.812, *p* < 0.05 ^b^ Δχ^2^ (1) = 2.784, *p* > 0.05 ^c^ Δχ^2^ (1) = 2.729, *p* > 0.05	Hypotheses testing: H8a: Supported H8b: Not supported H8c: Not supported		Goodness-of-fit statistics for the baseline model: χ^2^ = 1699.560, *df* = 806, *p* < 0.001, χ^2^/*df* = 2.109, RMSEA = 0.059, CFI = 0.902, IFI = 0.903, TLI = 0.894	
^d^ Δχ^2^ (1) = 0.158, *p* > 0.05	H8d: Not supported			
^e^ Δχ^2^ (1) = 0.009, *p* > 0.05	H8e: Not supported			
^f^ Δχ^2^ (1) = 0.123, *p* > 0.05	H8f: Not supported			
^g^ Δχ^2^ (1) = 2.998, *p* > 0.05	H8g: Not supported			

* *p* < 0.05,

** *p* < 0.01.

## Discussion and implications

This study helps us understand the impact of the perceived risk of PM, which threatens mankind due to rapid economic growth and industrialization, on desires and behavioral intentions through attitude, norms, and perceived behavior control. To accomplish the goal of this study, an empirical analysis was conducted. Specifically, the perceived PM risk was classified into physical, psychological, financial, functional, and time risks and the effect on three constituent variables of Ajzen's (22) verified TPB was investigated. It also examined the effect of attitude toward the behavior, subjective norms, and perceived behavioral control on desire and the effect of desire on behavioral intention. To check the difference in the perception of PM risks according to nationality, the moderating role was also verified for Chinese and Koreans, the countries most frequently exposed to the risk of PM concentration.

The results of analyzing the eight hypotheses presented in this study are as follows. First, out of the five risk factors of physical, psychological, financial, functional, and time risks presented as sub-components of the perceived risk of PM, people had a high perception of all the proposed risks. In particular, people perceive the greatest time risk when exposed to high PM levels. These results suggest that people perceive more diverse risks in addition to the health (physical) risks caused by PM, which previous studies have focused on. Therefore, this result shows the need for a more diverse approach to the perception of risk for PM and a specific approach to its potential negative impact. In addition, these results indicate the importance of these clues to improving the understanding of the risk and behavioral responses of people living in areas with high PM concentration levels.

Next, the perceived risk of PM was found to have a significantly negative effect on attitude toward the behavior, subjective norms, and perceived behavioral control. These results are consistent with the previous studies (55–57, 59) that the perception of risks influences an individual's attitudes, norms, and behavior, thereby influencing behavior intention. That is, an individual's perception of risk toward the PM leads to a change in their attitude to the behavior. It also influences how well they can perform and control the behavior and the subjective norms of social pressures for implementing or not implementing the behavior. Therefore, this study confirms that risk perception of PM can predict individual behavior, norms, and will, and that individual behavior is formed through the interaction of such perception with the above factors. In addition, it can be seen that when individuals perceive the various risks of high-level PM, they can increase their desire and intention to perform specific actions while reducing psychological distance.

As a result of the verification of hypotheses 4, 5, and 6 presented based on the verified TPB, it was found that individual attitudes, norms, and perceived behavioral control on the impact of PM risk had a significantly positive effect on individual desires. This result supports Hamid and Bano (57), which showed that individual risk perception changes attitudes and increases the desire to actively participate in preventive actions according to self-efficacy. This study indicates that the more people are aware of the dangers of PM, the greater their desire to refrain from environmentally harmful behaviors such as the use of individual vehicles, the use of fossil fuels, and the overuse of energy. Therefore, it is necessary to recognize the various problems and risks that PM can cause and implement policies to reduce PM, such as Carbon Neutral by 2050 and ESG principles, more actively. In addition, it will be necessary to raise people's awareness of the various problems that PM can cause and prepare a plan to address them actively.

In this study, to test hypotheses H8a–H8g in the proposed concept on the individual's behavioral intention through the risk perception of PM, the moderating role of nationality (China and Korea) was examined. A significant difference can be seen. Specifically, hypotheses H8b–H8g based on the present structural model did not reveal any appreciable differences in moderating effects according to nationality. However, nationality was found to play a statistically significant moderating role in the relationship between the perceived risk of PM and attitude toward the behavior. These results show that there is no significant difference between Chinese and Korean citizens in norms, behavioral control, desires, and behavioral intentions around PM risk perception, but that there may be differences in attitude. In fact, according to IQAir's 2021 World Air Quality Report (79), the average annual PM2.5 concentration (μg/m^3^) was found to be 32.6 for Mainland China and 19.1 for Korea. However, according to the results of this study, Korean (−0.847) and Chinese (−0.706) attitude formation varied according to PM risk perception. This result indicates that Koreans react more sensitively when it comes to attitude formation, even though China has a higher level of PM2.5 concentration. In addition, although no statistically significant difference was found in the verification of the moderating effect according to the suggested nationality, Koreans responded more sensitively than Chinese in most of the relationships ranging from risk perception and behavioral intentions with respect to PM. These results are theoretically very meaningful, as the attitudes can appear to differ depending on nationality regardless of the actual PM concentration.

The following theoretical and practical implications can be derived from the results of this study. The impact of PM on humans has already become a global concern, and thanks to government agencies and various media, people are aware of the risks of PM. However, both people's perceptions and existing studies of PM focus on risks to the human body and human activity (2–6, 8). As the results of this study indicate, people perceive a greater variety of risks due to PM, and even to that extent, the perception of time risk is higher than that of physical risk. Therefore, this study is significant because it expanded the scope of the existing perceived risk of PM, suggested more diverse risk factors, and established significance in PM research. In addition, it has verified its importance by applying the previously verified TPB of Ajzen (22) to the perceived risk of PM. Through this result, the research present very significant academic implications that the perceived risk of PM can change norms and beliefs about individual behavior and negatively affect control beliefs about the consequences of certain behaviors. Especially the environmental consequences. In addition, through the verification of the indirect effect, the three sub-factors of TPB mediate the perceived risk of PM, negatively affecting desire and behavior intention. This result provides additional academic implications for the mediating role of TPB.

The practical implications of this study are as follows. When people are exposed to the risk of PM, they become aware of its severity and respond accordingly. Specifically, when the concentration of PM is high, people refrain from activities or behaviors that can worsen air quality through individual norms and perceived behavior control and seek ways to improve it. However, the fundamental principle is to minimize exposure to the risks caused by PM. Therefore, the government should devise a way to reduce the use of solid fuel, the main source of PM, through an international agreement to reduce PM, and companies should also make a gradual effort to switch from using solid fuel to alternative energy. If these efforts are combined with periodic education and environmental improvement campaigns for the citizens of those countries where PM occurs, the frequency of exposure to PM risks will be minimized, and the quality of life will be improved. In other words, premature death and health loss due to disease can be reduced by reducing the risk of disease occurring due to PM. Protecting health, improving quality of life, and living longer and healthier can also reduce the various risks (e.g., physical, psychological, financial, functional, and time) to society.

## Conclusion

Globally, the risk of PM is an issue that cannot be overlooked. In particular, Northeast Asian countries are exposed to the risk of PM from yellow dust or solid fuel use. Therefore, to confirm the risk perceived by people due to PM, this study presented the physical, psychological, functional, financial, and time risks of PM through a literature review; examined the components of the verified TPB (e.g., attitudes toward behavior, subjective norms, perceived behavioral control); and confirmed their effect on individual desires and behavioral intentions. This study's results confirmed that people perceive risk for PM in various forms and that risk perception toward PM has a negative effect on attitude toward the behavior, subjective norm, and perceived behavioral control. In addition, the indirect effect verified that the above perception of risk has a negative effect on individual desires and behavioral intentions. Moreover, verified that there might be significant differences according to nationality in forming attitudes toward PM. Especially this study found that the perception of PM risk may differ depending on nationality or culture, rather than on actual statistical figures. Therefore, the purpose of this study was successfully achieved, and the results provided a theoretical and practical implication for the impact of PM risk perception on desire and behavior intentions have been presented.

## Limitation

This study has some limitations despite the significant findings. First, the results of this study on the perception of PM risk and behavior intentions have limited generalizability because the study targeted only citizens of two Northeast Asian countries with severe PM concentrations. Second, although the perceived level of risk may differ depending on the frequency of exposure to PM, one problem is raised that did not reflect this aspect of the study. Therefore, it is suggested that future research consider the role of nationality, cultural characteristics, the concentration level of PM, and frequency of PM throughout the year, in forming behavior intentions according to the risk perception of PM.

## Data availability statement

The original contributions presented in the study are included in the article/supplementary material, further inquiries can be directed to the corresponding author.

## Ethics statement

Ethical review and approval was not required for the study on human participants in accordance with the local legislation and institutional requirements. The patients/participants provided their written informed consent to participate in this study.

## Author contributions

All authors contributed to conceptualization, formal analysis, investigation, methodology, and writing and editing the original draft.

## Conflict of interest

The authors declare that the research was conducted in the absence of any commercial or financial relationships that could be construed as a potential conflict of interest.

## Publisher's note

All claims expressed in this article are solely those of the authors and do not necessarily represent those of their affiliated organizations, or those of the publisher, the editors and the reviewers. Any product that may be evaluated in this article, or claim that may be made by its manufacturer, is not guaranteed or endorsed by the publisher.
